# Postmortem computed tomography of gas gangrene with aortic gas in a dialysis patient

**DOI:** 10.1007/s13730-020-00456-y

**Published:** 2020-04-22

**Authors:** Rin Asao, Kazumasa Nishida, Hiromichi Goto, Yoshikazu Goto, Noriatsu Ichiba, Isao Ohsawa

**Affiliations:** 1 Department of Nephrology, Saiyu Soka Hospital, 1-7-22 Matsubara, Soka, Saitama Japan; 2 Department of Neurosurgery, Saiyu Soka Hospital, Soka, Japan; 3 Department of Radiology, Otsu City Hospital, Otsu, Japan

**Keywords:** Gas gangrene, Postmortem imaging, Dialysis, Intravascular gas, *Clostridia species*

## Abstract

Recently, postmortem imaging is sometimes used as an alternative to conventional autopsy. However, there are few case reports of postmortem imaging of dialysis patients. Here, we report a fatal case of gas gangrene involving a 76-year-old man who underwent dialysis. He died suddenly before a diagnosis could be established. Immediately after his death, postmortem computed tomography (PMCT) revealed gas accumulation in his right upper extremity and ascending aorta. Gas gangrene progresses rapidly and may sometimes result in sudden death before it is diagnosed. In this case, PMCT findings were useful to diagnose gas gangrene. Intravascular gas is a common finding on PMCT and is generally caused by cardiopulmonary resuscitation and decomposition. However, the detection of gas in the ascending aorta by PMCT was not described previously. Moreover, Gram stain and culture of the exudate showed anaerobic Gram-positive bacilli which suggested that the gas generation in the blood was caused by *Clostridia* species. To the best our knowledge, this is the first report of a dialysis patient whose cause of death was determined as gas gangrene using PMCT.

## Introduction

Gas gangrene is a necrotic infection of the soft tissue and muscles that progresses rapidly and it has high mortality rates. In approximately 80% of the cases, gas gangrene is caused by *Clostridium perfringens* which is a Gram-positive spore-forming bacillus commonly found in the soil and intestines of humans and animals [1, 2]. Deep penetrating injuries compromise the blood supply and create an anaerobic environment that is ideal for spore germination and bacterial proliferation [3]. Other predisposing conditions are bowel and biliary tract surgery, intramuscular injection, gynecologic procedure, and intrauterine fetal death [1]. Although the clinical course of *Clostridia* gas gangrene is fulminant, patients do not usually exhibit specific or severe symptoms at onset. In addition, early diagnosis of necrotizing infections may be confounded by other factors [1]. Crescendo pain is the most important clinical manifestation of such infections. However, because pain can be absent in patients receiving analgesic agents and those having diabetes-related neuropathy or altered mental status, patients sometimes die shortly before a definitive diagnosis is established [1].

Currently, postmortem imaging is sometimes used as an alternative to conventional autopsy. In Japan, approximately 40,000 conventional autopsies were performed every year in the 1980s. However, the reported number of conventional autopsies is gradually decreasing. In the 2010s, the number of conventional autopsies was slightly higher than 11,000 per year [4]. Postmortem imaging has a shorter examination time and is less invasive than conventional autopsy. Wagensveld et al. evaluated the frequency of total-body computed tomography (CT) and magnetic resonance imaging features of postmortem changes in in-hospital deaths and reported a wide variety of features of postmortem changes [5]. To investigate the accuracy of postmortem imaging, a few groups compared postmortem imaging with conventional autopsy and showed the accuracy of postmortem computed tomography (PMCT) [6, 7]. PMCT is useful in detecting gas accumulation inside the body which is an advantage over the conventional autopsy. Therefore, PMCT is expected for a diagnosis of gas gangrene. Recently, few reports described PMCT with gas gangrene. Yamaguchi et al. reported the first autopsy case of fatal septicemia caused by gas-forming *C. perfringens* using PMCT findings. They combined conventional autopsy and PMCT to investigate a cadaver which was presented to the hospital in a cardiopulmonary arrest state and died [8]. Kitano et al. reported a case of in-hospital death in which PMCT revealed sepsis to be the cause of death [9].

To the best of our knowledge, there are few reports which describe PMCT in dialysis patients. Here, we report a dialysis patient who died suddenly owing to gas gangrene. In this case, we determined the diagnosis by PMCT. Additionally, we detected an intriguing finding, aortic gas.

## Case report

A 76-year-old man, who was diagnosed with type 2 diabetes mellitus (DM) 26 years ago, underwent dialysis for 9 years owing to diabetic nephropathy. He walked into a hospital at 22:00 p.m. with complaints of fever and abdominal discomfort. The medical history of the patient included cerebral infarction but not infection. He was awake and had a blood pressure of 189/94 mmHg, pulse of 102 beats per minute, body temperature of 38.8 °C, and saturation of percutaneous oxygen of 98% at room air. Physical examination showed that he had upper abdominal tenderness with normal bowel sounds. He had left incomplete paralysis owing to prior cerebral infarction. He did not complain of pain in his extremities and no apparent injury was found, except for a vascular puncture site for a shunt in his left forearm. Laboratory test results revealed a mildly elevated leukocyte count (11,600/${\upmu }$L), and his DM was poorly controlled (Table 1). The systemic inflammatory response syndrome score met two criteria (body temperature > 38 °C and heart rate > 90/min). X-ray, electrocardiogram, and chest and abdominal CT showed no remarkable alterations. Based on the physical examination and laboratory tests, enterogastritis was initially suspected. However, he complained that his pain worsened and experienced sudden swelling in his right upper extremity at 7:00 a.m. Despite cefazolin administration, he rapidly developed a state of shock and had a cardiopulmonary arrest at 7:50 a.m. Cardiac compression and artificial respiration with bag valve mask and respirator were performed for 30 min. Central venous catheterization was not performed. Despite attempts of resuscitation, his death was confirmed.

**Table 1:** Laboratory findings on admission

WBC	11,600/µL	AST	13 U/L	Na	139 mmol/L
RBC	390 × 10^4^/µL	ALT	9 U/L	K	4.2 mmol/L
Hb	13.2 g/dL	γGTP	17 U/L	Cl	103 mmol/L
Ht	39.40%	ALP	192 U/L	Ca	9.3 Mg/dL
Plt	11.8 × 10^4^/µL	LDH	162 U/L	HbA1c	7.8%
PT	11.7%	CK	99 U/L	CRP	0.17 mg/dL
APTT	26.8 s	T-Bil	0.7 mg/dL		
		BUN	46.1 mg/dL		
		Cr	8.22 mg/dL		

**Fig. 1:**
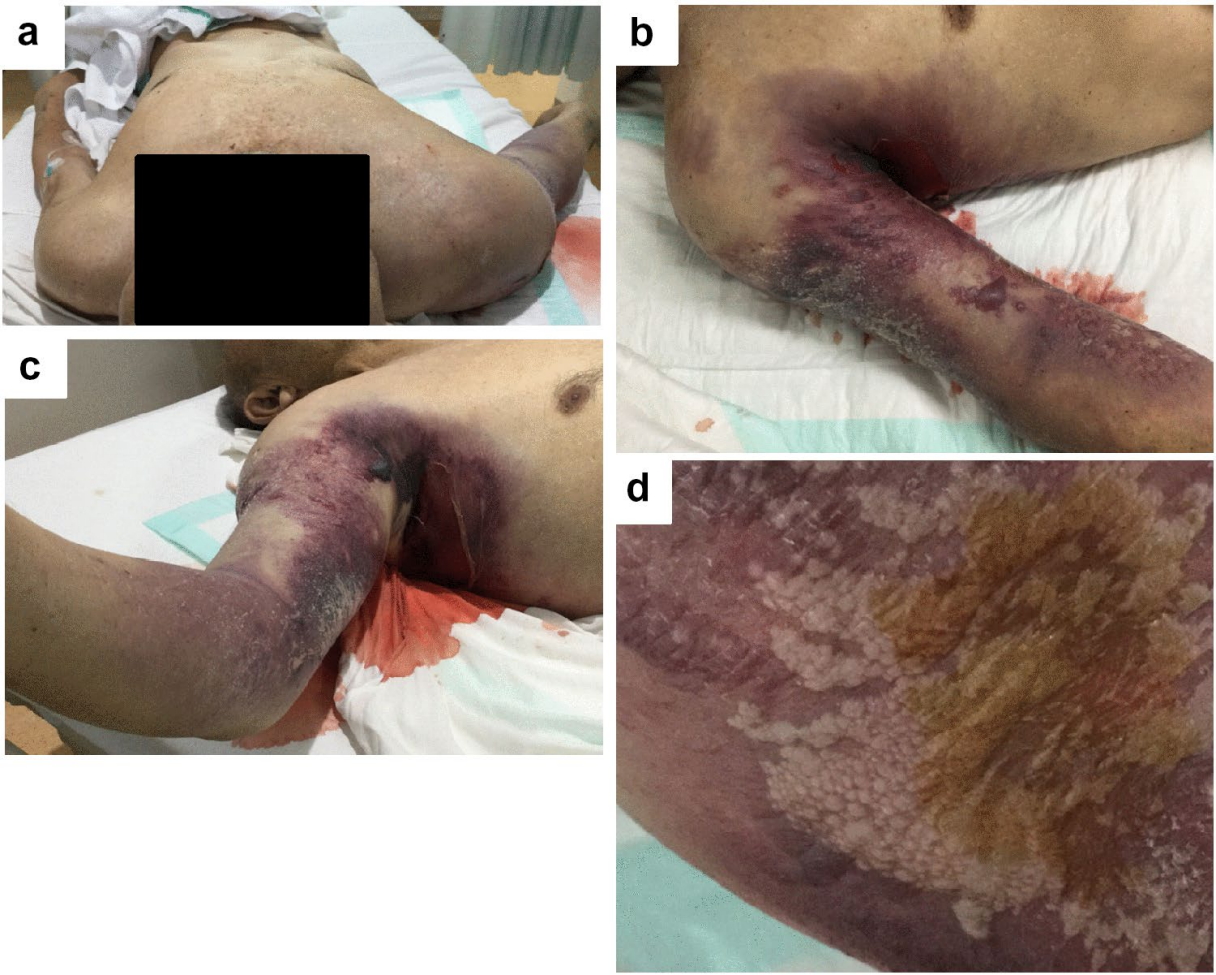
External appearance: **a** skin purpura and swelling on the torso and right upper extremity. **b**, **c** Cutaneous emphysema and subcutaneous hemorrhage on the right upper extremity. **d** Bubbles in skin blisters

The cadaver had cutaneous emphysema and subcutaneous hemorrhage on the torso and right upper extremities (Fig. 1a–c). Moreover, the deceased patient developed skin blisters with a foul smell (Fig. 1d). Because his family refused a conventional autopsy, whole-body PMCT was performed within 70 min of his death. A 64-multidetector-row CT (SOMATOM Perspective, Siemens Healthineers, Germany) scan was performed. The patient was scanned from the head to the pelvis in the supine position.

**Fig. 2:**
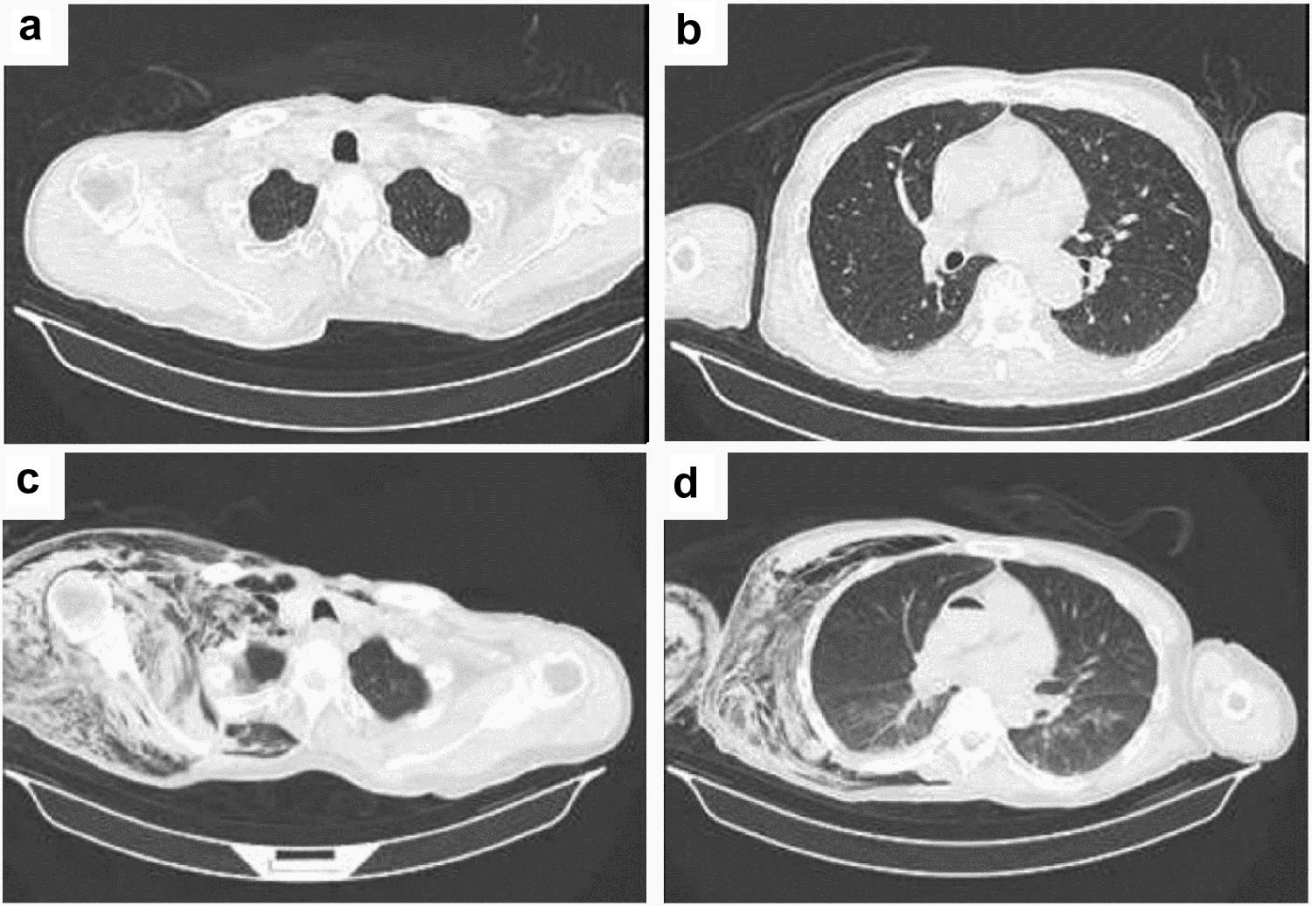
Chest computed tomography images on admission (upper panels) and postmortem computed tomography images (lower panels): **a** no remarkable changes were observed in subcutaneous regions and muscles. **b** No remarkable changes were observed in lung and cardiovascular system. **c** A massive amount of gas was observed in the right upper extremity. **d** Accumulation of gas was observed both within the torso and in the aorta

Although chest CT on admission showed no gas densities in the right upper extremity (Fig. 2a, b), PMCT showed remarkable gas densities in the subcutaneous and muscular layer of the right chest wall and upper extremity (Fig. 2c). Moreover, gas was observed even in the ascending aorta (Fig. 2d). A blood culture could not be performed owing to technical problems. A culture test result of the exudate from the skin bullae revealed a proliferation of Gram-positive bacilli. No aerobic bacteria were detected. We presumed that the cause of death was gas gangrene caused by *Clostridia* species.

## Discussion

Gas gangrene consists of two major presentations: traumatic and spontaneous. Traumatic gas gangrene occurs after crush-type injury, penetrating injury, gynecologic surgery such as abortion, and bowel/biliary tract surgery where bowel contents leak into the soft tissues. Spontaneous gas gangrene generally occurs via hematogenous seeding of bacteria from the gastrointestinal tract. However, in approximately 20–30% of the cases, the primary source of the infection is unknown [10, 11]. In this case, because the patient had no apparent traumatic injury, except for a vascular access puncture site for a shunt in the left forearm, and initially complained of abdominal discomfort, the focus and route of this infection are unknown. A very small injury may have existed in his upper right extremity. *Clostridia* species, *E. coli*, *Klebsiella pneumoniae, Aeromonas hydrophilia,* etc. are known as pathogens that cause gas gangrene [1, 9, 11−13]. In this case, Gram staining of the exudate from the skin bullae showed Gram-positive bacilli. Thus, it was conceivable that the gas gangrene was caused by *Clostridia* species.

When interpreting PMCT images, normal postmortem changes should be recognized and distinguished from fatal findings. Intravascular gas is a common finding on PMCT, and two mechanisms were proposed for gas development: cardiopulmonary resuscitation (CPR) [14−19] and decomposition [15, 20]. Decomposition of gas occurs within 24–48 h of postmortem and is typically produced by intestinal flora [15, 20]. Chest compression during CPR causes the vaporization of dissolved gas in blood [15, 17]. The veins are more likely to collapse and expand in response to CPR than arteries, and as a result, dissolved gas in the blood tends to vaporize [21]. Furthermore, CPR induces the rupture of pulmonary vessels combined with lung parenchymal destruction that permits air to enter the pulmonary vein and reach systemic circulation [17−19]. Cardiovascular gas was observed in 71% of the patients who underwent CPR and gas accumulation was observed in the right heart in most cases. However, there is no reported case of aortic gas development [17]. In this case, intravascular gas was observed not in the right heart but in the aorta. Okuda et al. reported the case of myocardial intravascular gas in the left ventricle on PMCT performed immediately after death certification [22]. As CO_2_ concentration in the right heart and myocardial vein increased because of low cardiac output, anaerobic metabolism progression, and decreased CO_2_ transport, the blood became supersaturated and released excess solute CO_2_ as gas bubbles after death [22]. Kitano et al. reported a case of death owing to sepsis without CPR. PMCT performed 2 h after death revealed intravascular gas [9]. They described that the cause of intravascular gas on PMCT was not only CPR and decomposition but also gas-producing bacterial sepsis [9]. In this case, PMCT was performed immediately after death certification, and intravascular gas was detected in the ascending aorta. Therefore, decomposition and CPR were less likely to be the cause of aortic gas than gas-forming bacteria. As *Clostridia* species produce large quantities of gas rapidly in an anaerobic condition, it was conceivable that aortic gas was produced by *Clostridia* species in this case.

This case report has some limitations. First, there was a lack of clinical data before the patient’s condition suddenly worsened. Because this patient had poorly controlled diabetes and underwent dialysis, it is likely that he was immunocompromised. Thus, there was a high prevalence of infection in this case. Second, pictures of Gram staining of the exudate were not taken and anaerobic culture of exudate and blood culture was not performed, although these tests were necessary to identify the pathogen causing gas gangrene. However, Gram staining and culture of the exudate showed anaerobic Gram-positive bacilli which suggested that the responsible bacteria might be *Clostridia* species.

In conclusion, we report a gas gangrene case that was diagnosed based on clinical course and PMCT findings. To the best of our knowledge, this is the first report of a dialysis patient whose cause of death was determined as gas gangrene using PMCT. Gas gangrene progresses rapidly and may sometimes result in sudden death before a diagnosis is made. Moreover, aortic gas on the PMCT performed immediately after the death of a patient with septicemia is a key finding of this report.
